# Structural and Functional Insights into the Pilotin-Secretin Complex of the Type II Secretion System

**DOI:** 10.1371/journal.ppat.1002531

**Published:** 2012-02-09

**Authors:** Shuang Gu, Saima Rehman, Xiaohui Wang, Vladimir E. Shevchik, Richard W. Pickersgill

**Affiliations:** 1 Queen Mary University of London, School of Biological and Chemical Sciences, London, England; 2 Université de Lyon, Université Lyon 1, Lyon; INSA-Lyon, Villeurbanne; CNRS, UMR5240, Microbiologie Adaptation et Pathogénie, Lyon, France; IMP/IMBA Research Center, Austria

## Abstract

Gram-negative bacteria secrete virulence factors and assemble fibre structures on their cell surface using specialized secretion systems. Three of these, T2SS, T3SS and T4PS, are characterized by large outer membrane channels formed by proteins called secretins. Usually, a cognate lipoprotein pilot is essential for the assembly of the secretin in the outer membrane. The structures of the pilotins of the T3SS and T4PS have been described. However in the T2SS, the molecular mechanism of this process is poorly understood and its structural basis is unknown. Here we report the crystal structure of the pilotin of the T2SS that comprises an arrangement of four α-helices profoundly different from previously solved pilotins from the T3SS and T4P and known four α-helix bundles. The architecture can be described as the insertion of one α-helical hairpin into a second open α-helical hairpin with bent final helix. NMR, CD and fluorescence spectroscopy show that the pilotin binds tightly to 18 residues close to the C-terminus of the secretin. These residues, unstructured before binding to the pilotin, become helical on binding. Data collected from crystals of the complex suggests how the secretin peptide binds to the pilotin and further experiments confirm the importance of these C-terminal residues *in vivo*.

## Author Summary

Pathogenic bacteria deliver toxins and virulence proteins into host cells and tissues using specialised secretion systems such as the type II and type III secretion systems. These secretion systems have a pore formed by secretin protein subunits through which the disease causing protein effectors and toxins pass. The secretin must be targeted to and assembled in the outer-membrane and a pilotin protein facilitates this process. In the absence of the pilotin the secretin is degraded or mislocates to the inner-membrane, in either case the secretion system in non-functional and the bacterium cannot cause disease. Here we show how the pilotin and the secretin of the type II secretion system interact, these insights may be useful for the development of antibacterial compounds to interfere with secretin targeting and assembly and defeat pathogenic bacteria such as *Vibrio cholerae* and enterotoxigenic *E. coli* which infect man and *Dickeya dadantii* which threatens food security.

## Introduction

The secretins are an important group of bacterial membrane proteins whose function is to facilitate the transport of secreted proteins and macromolecular complexes across the outer membrane [1].They are essential components of the type II and type III secretion systems (T2SS and T3SS respectively) and play a key role in the assembly of type IV pili (T4P) and release of filamentous bacteriophages. Determination of the structure of secretins has been confined to low-resolution transmission electron microscopy and cryo EM studies [2]–[5] which show membrane penetrating ring structures with 12–14 fold rotational symmetry. A specialized class of small lipoprotein pilotins bind their cognate secretins and facilitate oligomerization, insertion and proper assembly in the outer bacterial membrane. In this paper we explore the structure and function of the pilotin from *D. dadantii* (OutS), several other pilot proteins have been described [6]–[12]. Pilotins whose structures have been determined are MxiM (PDB code: 1Y9L) of the T3SS of *Shigella flexneri* and PilW/PilF (PDB codes: 2VQ2)/2FI7 and 2HO1) of the T4P of *Neisseria meningitidis* or *Pseudomonas aeruginosa* [13], [14]. The cracked β-barrel structure of MxiM has been solved in complex with an 18 residue peptide from the cognate secretin MxiD (PDB code: 2JW1) and the authors propose a model for the way MxiM assists MxiD assembly [10], [15]. The other known pilot structure, PilW/PilF, appears to perform a broadly similar function to MxiM, ensuring multimerization of the secretin PilQ into the outer membrane, but has a different architecture comprising six serial α-helical tetratricopeptide repeats [9], [12]. A third auxiliary secretin-binding protein has been characterised structurally, PilP, which is also involved in the assembly or stability of the secretin PilQ of *Pseudomonas aeruginosa* [16], the structure comprises a sandwich of two sheets each with three anti-parallel β-strands.

The type II secretion system spans both the inner and outer bacterial membranes [17], [18]. It consists of an inner membrane subcomplex, periplasmic pseudopilins and the outer membrane secretin [19]. There have been considerable recent advances in our understanding of the T2SS secretin. First the structure of the N-terminal periplasmic domains N0, N1, and N2 in complex with a nanobody [20] was elucidated (PDB code 3EZJ) and secondly a cryo EM reconstruction of the secretin itself has been described [3]. In the absence of pilotin the *D. dadantii* secretin (OutD) mislocates to the inner membrane [21]. The pilotin possesses at its N-terminus the characteristic lipoprotein signal sequence (LAAC), with the signal peptidase LspA cleaving site just before the cysteine to which the lipid is covalently attached. The pilotin (OutS) binds to the C-terminal 62 residues of the secretin (OutD) [11], [19]. Here we elucidate the structure of the T2SS pilotin and show that it binds tightly to 18 residues close to the C-terminus of the secretin subunit causing this unstructured region to become helical on forming the complex.

## Results/Discussion

### Structure of the T2ss Pilotin

**Figure 1:**
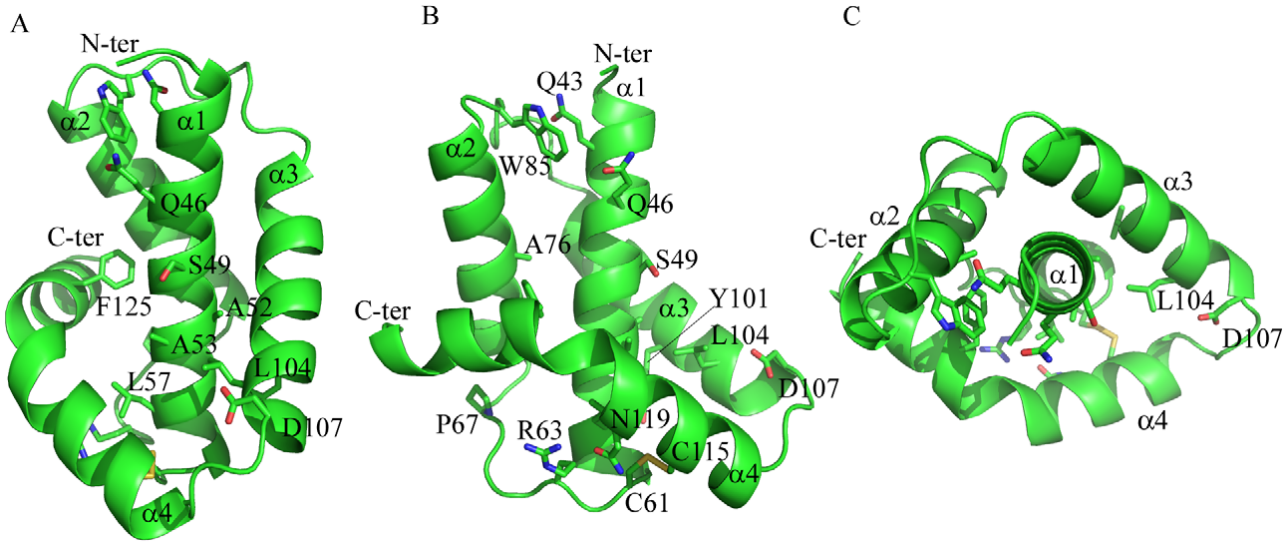
Structure of the pilotin. The crystal structure of *Dickeya dadantii* OutS consists of four α-helices, the last of which is bent. (A) α3 and bent α4 wrap around the anti-parallel hairpin formed by α1 and α2. Conserved residues (see Figure S1 for sequence alignment) are represented as sticks. (B) Rotated about the y-axis by 90°, this view reveals the concave surface formed between helix α1 and helices α3and α4. The disulfide can be seen linking α1 and α4. (C) Rotated around the x-axis by 90°, α1 is surrounded by the other three helices. A52, A53, S49 and D107 are not strictly conserved but the first three are always small and 107 is either D or E suggesting some functional constraint on this region. Q46 is absolutely conserved and may map the other extremity of the binding site. Figure 1 and panels D and E of Figure 3 were produced using PYMOL.

To ensure authentic folding and production of soluble *Dickeya dadantii* pilotin (OutS) in the *E. coli* periplasm, the PelB secretion sequence was substituted in place of the N-terminal lipidation sequence thereby preventing lipidation of the pilotin. This substitution facilitated protein production and crystallization without compromising secretin-binding [11]. Cleavage of the secretion signal accompanies transport in to the periplasm. The crystal structure of the pilotin was determined using the anomalous scattering from a potassium tetrachloroplatinate derivative and the structure refined at 1.65 Å resolution (Table 1). The two copies of the pilotin subunit in the asymmetric unit of the crystal are virtually identical in structure (root mean square deviation of 94 α-carbon atoms, residues 38 to 132, is 0.243 Å). The structure is clearly defined in the electron density map except for the N-terminal residues preceding Val 38 which presumably form a flexible linker to the lipidation site. The architecture of the pilotin is the remarkable insertion of one α-helical hairpin into a second open α-helical hairpin with bent final helix (Figure 1); this is unlike the two other pilotin structures solved and is profoundly different from any previously described four helix bundle. The first helix of five turns (residues 40–60), is connected to the second of four turns (residues 69–82) by an 8 residue loop. The second loop of 10 residues connects to the third helix of four turns (residues 93–106) which packs against helix one. A short three residue loop which connects helices three and four and the disulfide bridge, between Cys 115 in the second turn of helix four and Cys 61 the first residue of the helix one to helix two loop (Figure 1B), sets the scene for the packing of helix four (residues 111–130). The pronounced bend of 65° in helix four is important for the architecture; the helix has three large hydrophobic residues which are at least partially buried by interactions with hydrophobic residues on the other three helices: Phe 118, Met 122, and Phe 125. The requirement to pack conserved Phe 125 appears to dictate the severe bend of this helical element. In the crystal the pilotin subunits form a dimer, with Arg 63 and Asn 119 (Figure 1B) involved in an electrostatic interface, between subunits, however there is currently no evidence that dimerization occurs in solution or *in vivo*.

**Table 1:** Crystallographic data and refinement statistics for pilotin.

*Data Collection*	Native	Tetrachloroplatinate derivative
Space group	P2_1_2_1_2_1_	P2_1_2_1_2_1_
Cell parameters (Å)	*a = 49.7, b = 53.1, c = 98.7*	*a = 49.8, b = 51.8, b = 97.9*
Molecules per asymmetric unit	2	2
Platinum sites/au	0	4
Wavelength (Å)	1.0718	1.0718
Resolution (Å)	46.78–1.65 (1.71–1.65)^a^	52.81–2.90 (3.06–2.90)^a^
Number of unique reflections	32440 (4652)^a^	6121 (873)^a^
Multiplicity	7.8 (6.8)^a^	12.6 (13.4)^a^
Completeness (%)	100.0 (99.9)^a^	99.9 (100.0)^a^
R_merge_ (%)^b^	0.200 (0.336)^a^	0.103 (0.214)^a^
Mean I/sigma (I)	6.9 (2.6)^a^	19.2 (12.3)^a^
R_pim_ (%)^c^	0.063 (0.140)^a^	0.033 (0.062)^a^
MSAN^d^	-	1.20
Wilson B-factor (Å^2^)	21.4	60.1
*Refinement*		
Resolution limits (Å)	46.8–1.65	
Reflections (work/test)	30555/1578	
R-factor/R-free^e^ (%)	0.197/0.249	
rmsd bond(Å)/angle (°)	0.006/0.918	
Number of protein (solvent) atoms	1465 (195)	
Average B-factor protein (solvent) (Å^2^)	30.9 (46.0)	
Ramachandran plot statistics (%)		
Residues in most favoured regions	98.4%	
Residues in additional allowed regions	1.6%	

a The parameter values for the range 1.85–1.76 Å and 3.06–2.90 Å are given in parentheses for native and heavy metal derivative data, respectively.

b R_merge_ = Σ_hkl_ Σ_i_|I_i_−<I>|/_Σ_hkl_ ΣI_i_, where I_i_ is the intensity of the i^th^ observation, <I> is the mean intensity of the reflection, and the summations extend over all unique reflections (hkl) and all equivalents (i), respectively.

c R_pim_ = Σ_hkl_ [n/(n−1)]^1/2^ Σ_i_|I_i_(hkl)−<I(hkl)>|/Σ_hkl_ Σ_i_ I_i_(hkl), where n is the multiplicity, other variables as defined for R_merge_ [46].

d MSAN is the Mid slope of Anomalous normal probability.

e R-factor = Σ_hkl_|F_o_−F_c_|/Σ_hkl_ F_o_, where F_o_ and F_c_ represent the observed and calculated structure factors, respectively. The R-Factor is calculated using 95% of the data included in refinement and R-free the 5% excluded.

### Sequence Similarity and Structural Similarity

The majority of the 13 absolutely conserved residues in the sequence alignment (Figure S1A) appear to be of structural rather than of direct functional significance. The two highly conserved cysteine residues, 61 and 115, form the disulfide bridge between helices α1 and α4 that stabilizes the correct nested α-helical protein fold of the pilotin is functionally relevant. When a reducing agent was used in pull-down assays or in bacterial two-hybrid tests, the pilotin was unable to bind the cognate secretin (Figure S6). Presumably this is because the disulfide is reduced and the pilotin did not fold correctly in the cytoplasm. Interestingly, the previous mutagenesis analysis revealed several structurally or functionally relevant residues of pilotin (OutS), notably conserved Leu 57, Arg 63 and Ser 97 [11]. Substitution each of these prevented secretin (OutD) targeting to the outer membrane. Conserved Cys 21 covalently attaches to the lipid and this residue is essential for targeting the pilotin to the outer membrane. Conserved Gln 46, on the solvent exposed surface of helix 1 (Figure 1), must also be of functional rather than structural significance and maps to the extremity of the concave surface of the pilotin formed by helices α1, α3 and α4; it is plausible that this concave surface is the binding site for the secretin (Figure 1B). Conserved residues including: Gln 46 and Leu 104 and semi-conserved Leu/Val/Ile 50, Phe/Leu 118 are also in this region. Residues 49, 52 and 53 on the solvent exposed surface of helix α1 are Ser/Ala for the former two and Ala/Gly for the latter, respectively (Figure S1A). This conservation of small residues at these positions is consistent with this region being important in binding as there is no structural reason why larger residues could not be accommodated at these sites unless the secretin binds tight up against the first helix (Figure 1). A DaliLite database search revealed that P40 nucleoprotein has a similar arrangement of α-helices to that of the pilotin. The Dali score was 6.0 and sequence identity 5%. The P40 nucleoprotein domain architecture is however substantially more complex with seven helices instead of the pilotin's four. The concave surface of the corresponding helices of P40 nucleoprotein is occupied by a helix supporting the view that this is the binding site for an α-helix. Mutations of the pilotin binding surface confirm the importance for binding of some of the residues decorating the concave surface (Table S1). Mutating Ser 49 to Arg has a profound effect on binding and the mutants Leu 96 Ala, Leu 100 Ala and Gln 114 Ala have a substantial effect on binding as expected if this is the binding surface (Figure 1B).

### Secretin-Binding to the Pilotin in Solution

**Figure 2:**
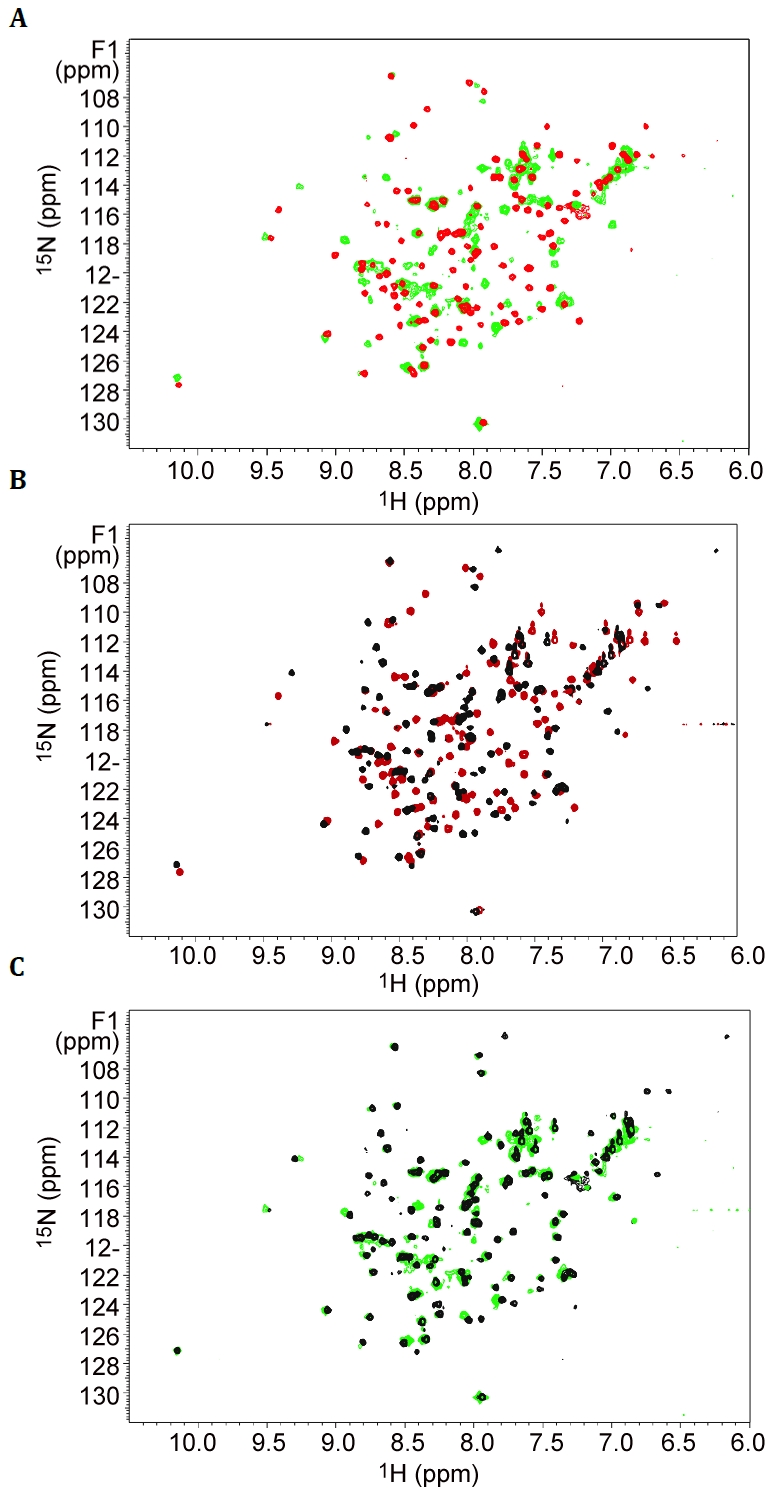
Elucidation of secretin-pilotin interactions. Titration of secretin peptides into ^15^N labelled pilotin. ^1^H-^15^N-HSQC spectra of the pilotin in the absence of secretin (red spectra), in the presence of 62 residue secretin peptide (green) and in the presence of 18 residue peptide (black). Protein concentration was 100 µM. (A) Pilotin in the absence and presence of 62 residue secretin peptide. (B) Pilotin in the absence and presence of the 18 residue secretin peptide. (C) Overlay of the two complexes with secretin peptides showing the 18 residue peptide is behaving in a closely similar way to the 62 residue peptide.

**Figure 3:**
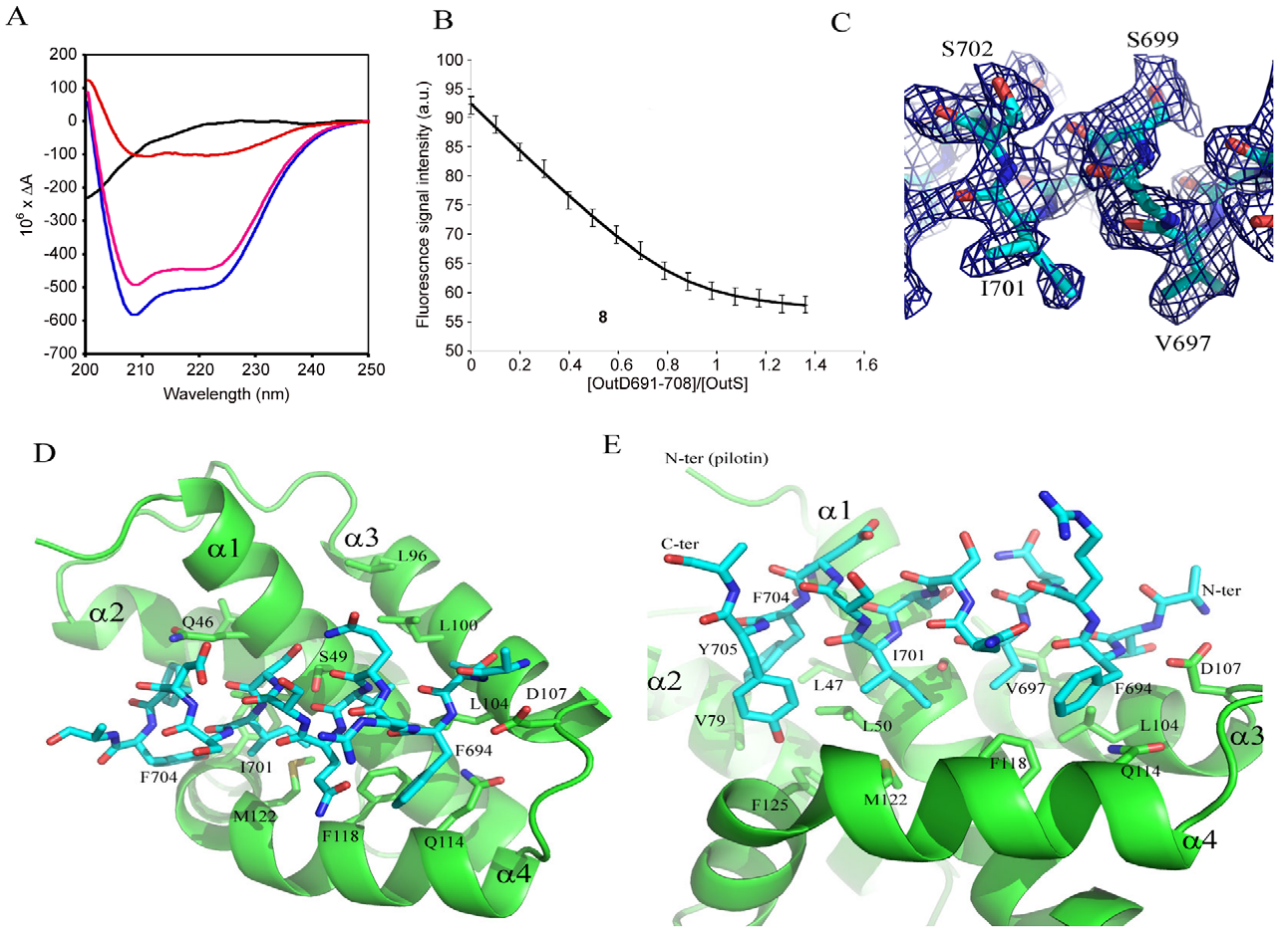
The secretin-pilotin complex. (A) The far uv circular dichroism spectra of the 18 residue secretin peptide alone (black), pilotin alone (pink) and a stoichiometric ratio of both pilotin (OutS) and secretin peptide (OutD^691–708^) together (blue). The difference between the secretin/pilotin complex and the pilotin only is shown in red. As forming the stoichiometric ratio diluted both proteins by half, these data were multiplied by two to compensate for the dilution factor. The concentration of both pilotin and secretin peptide were 0.55 mM. 3JHNHA evidence of helical conformation is presented in Figure S5. (B) Measurement of the binding affinity of the pilotin OutS for the 18 residue secretin peptide determined using fluorescence spectroscopy. 1 µM pilotin was titrated with 50 µM secretin peptide in to 1 µM pilotin so that there was no dilution of pilotin. The stoichiometry can be seen to be 1∶1 within experimental error. The *K_d_* is 55±20 nM. Details of the equation fitted can be found in Table S1. (C) Part of the simulated annealed omit map showing the quality of the electron density map used to derive the models shown in (D) and (E). (D) Model of the secretin peptide bound to the pilotin, D107 acts as an N-terminal helical cap. (E) Close up showing the hydrophobic nature of the complementary side chains involved in forming the complex.

It had previously been shown that the C-terminal 62 residues of the secretin bound to the pilotin [11]. This secretin peptide was produced with ^15^N-label as a fusion to GST and then released with PreScision protease. The backbone amide protons were poorly dispersed in the ^1^H-^15^N HSQC spectra revealing the C-terminal 62 residues are unstructured in solution (Figure S4). NMR cross-titration studies revealed that only peaks corresponding to residues 691–708 of the secretin peptide were shifted on addition of unlabelled pilotin (Figure S2; with assignment of secretin peptide shown in Figure S3). When the pilotin was ^15^N-labelled, good dispersion of the backbone amides protons was observed as expected given its folded structure (Figure 2). Titration of the unlabelled 62 residue secretin peptide into the ^15^N-labelled pilotin produced a large number of peak shifts (Figure 2A). Shift perturbations are extremely sensitive indicators of structural changes and the extent of the changes observed is compatible with the secretin peptide decorating the surface of the pilotin and causing subtle structural rearrangements, perhaps in packing interactions in the hydrophobic core, reflected in chemical shift changes across much of the structure. The secretin peptide binds to the pilotin in a 1∶1 stoichiometric ratio as determined from the NMR titration where the intensity of the shifted peaks of the complex saturate at an equimolar ratio of OutD to OutS. Since only residues 691–708 of the secretin were affected by interaction with pilotin (Figure S2), a synthetic 18 residue peptide corresponding in sequence to these residues was assessed. The pattern of shifts in ^1^H-^15^N HSQC pilotin spectrum observed using this synthetic 18 residue peptide was identical to that using the 62 residue secretin peptide (Figure 2B, C) confirming that it is these 18 residues that are those principally involved in binding to the pilotin. Circular dichroism measurements also showed the unstructured nature of the secretin peptide and provided evidence that the peptide becomes helical on binding (Figure 3A). The signal saturates at a stoichiometric ratio of secretin peptide to pilotin. The CD spectra of the pilotin and secretin peptide together correspond to more helical structure than the spectra of the pilotin and secretin peptide summed. The additional helical content can be quantified as 12 residues assuming all secretin and pilotin molecules are in complex, a reasonable assumption given the high affinity of complex formation (see below). The most plausible explanation for this is that 12 residues of the secretin peptide become helical on binding to the pilotin. The helical propensity of the 18 residue secretin peptide was apparent from secondary structure predictions (Jpred [22] and shown on Figure S1B). To estimate the binding affinity of pilotin for the secretin peptide, fluorescence spectroscopy was used. Since the 18 residue secretin peptide has no tryptophan residues, quenching of the fluorescence signal from the single tryptophan residue in the pilotin on addition of the secretin peptide, was used to determine the affinity of binding. The 1∶1 stoichiometric binding ratio can be seen from the saturation of the fluorescence quenching of OutS by an equimolar quantity of the secretin peptide (Figure 3B). The binding of the 18 residue peptide is tight with *K*_d_ of 55±20 nM (Figure 3B) and is comparable to that of the T3SS pilotin-secretin complex [10], [15]. 3JHNHA spectra of the complex showed peaks coupled by less than 5 Hz (Figure S5) confirming that at least four residues of the secretin peptide become helical on binding to the pilotin, there may be more, but they are hidden by overlapping peaks.

### Model of the Pilotin/Secretin Peptide Complex

Crystals of the pilotin/secretin complex were grown, they belong to space group P6_5_ but are twinned (Table S2) and have solvent channels with disordered density within. Nevertheless four pilotin subunits can be found by molecular replacement and density for peptide can be seen occupying the concave surface of the pilotin. The evidence that suggests residues 694 to 704 of the secretin adopt a helical conformation in the electron density map, a part of the simulated annealed omit map is shown in Figure 3C. Ten residues are in helical conformation and the hydrophobic surface of the amphipatic helix interacts with the hydrophobic surface of the pilotin (Figure 3D, E). In this model the methyl groups of T692 and V697 interact with L100 and L104, I701 interacts with L50 and M122; F704 (with L47, V79 and F125) and F694 (with F118, L104 and Q114) interactions occur either side of these central interactions. D107 is the N-terminal helix capping residue, stabilizing the helix dipole of the bound secretin peptide. The peptide binds tight up against Q46, S49, A52 and A53, providing an explanation for their conservation or presence only as small residues in the case of the latter three. Interestingly, the same dimer as described for the non-complexed secretin is seen in these crystals too suggesting that the interaction may have some biological relevance. The quality of the refinement is relatively poor because of the disordered protein and for that reason the structure is being referred to as a model of the secretin peptide/pilotin interaction.

Intrinsically disordered regions of proteins such as these C-terminal residues of the secretin subunit facilitate binding by increasing their capture radius for cognate partner, the so-called fly-casting mechanism [23]. Initial weak binding may draw the secretin and pilotin together and as the secretin peptide folds on the pilotin surface the binding becomes tighter, locking the two together.

### Assessment of the Pilotin/Secretin Interaction In Vivo

**Figure 4:**
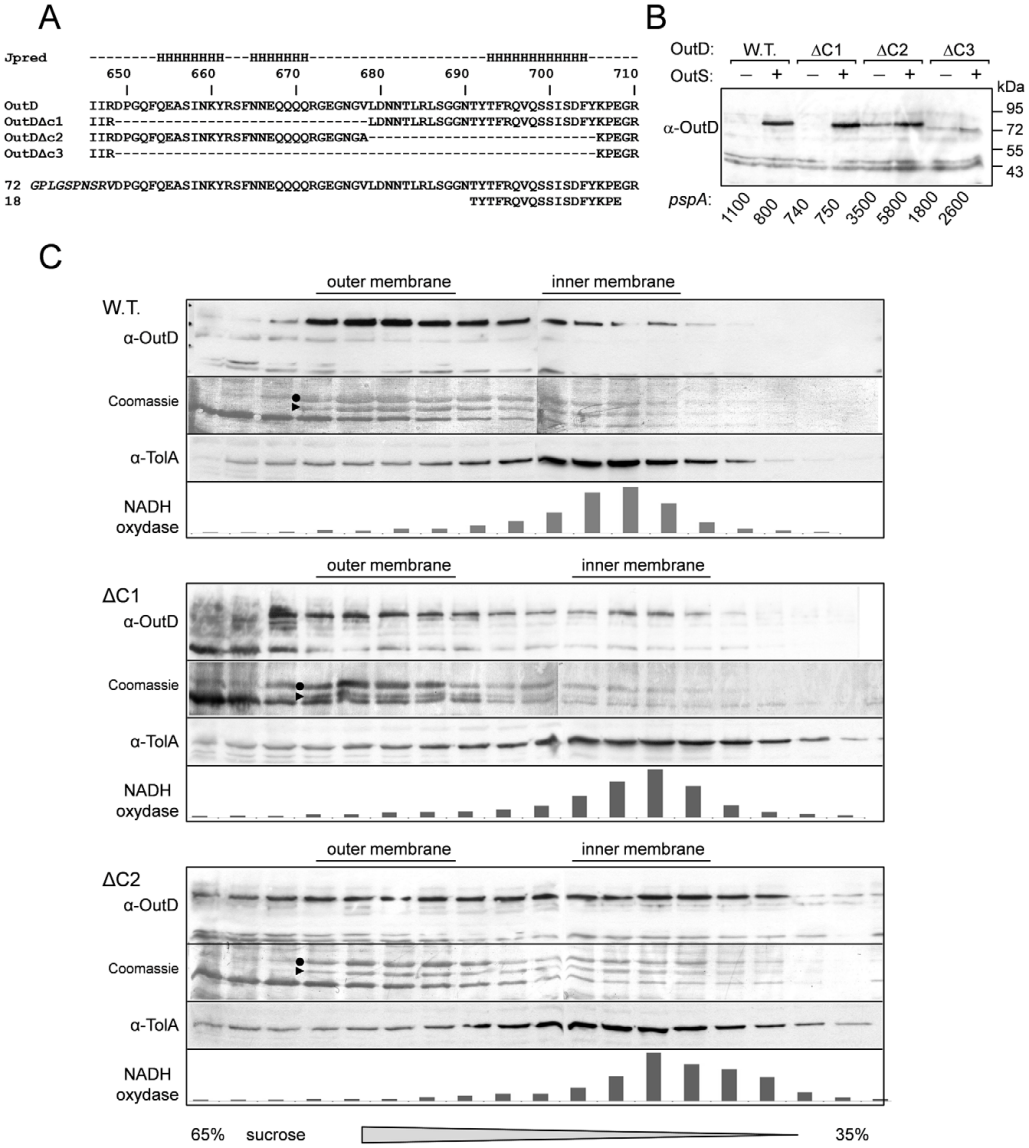
*In vivo* determination of secretin interactions with pilotin. Removal of the extreme C-terminal region of the secretin results in mislocalization of the secretin to the inner membrane (A) Sequence of the C-terminus of *Dickeya dadantii* secretin (OutD) along with the secondary structure prediction for this region; the predicted three helices are marked “H”. The sequences of the three secretin deletion mutants (OutDΔC1 to OutDΔC3) and the C-terminal secretin peptides used are shown. (B) Stabilization of truncated derivatives of the secretin (OutD) by pilotin (OutS) *in vivo*. *E. coli* MC3 cells expressing an OutD derivative (indicated above) and either OutS (+) or empty pACT3 vector (−) were grown for 12 h at 30°C in LB medium and then analyzed by immunoblotting with OutD antibodies. In the same cultures, β-galactosidase activity was assessed to estimate expression of *pspA-lacZ*. An elevated level of *pspA* reflects mislocalization of the corresponding secretin derivative to the inner membrane. Equivalent amounts of cell extracts were loaded into each well and used for activity measurement. (C) Pilotin promotes the outer membrane location of the full-length secretin OutD and truncated secretin OutDΔC1 but not OutDΔC2. The whole membrane fraction from *E. coli* NM522 cells coexpressing the indicated secretin derivatives and pilotin was separated by flotation sucrose gradient centrifugation and analyzed by immunoblotting with OutD- antibodies or stained with Coomassie G-250 to detect the major porins, which reflect the position of the outer membrane. Immunoblotting with TolA-antibodies and NADH-oxydase activity indicate the position of the inner membrane fractions. OmpA is indicated by a triangle and OmpC/F by a dot.

A series of *in vivo* experiments were used to test the proposed model of secretin binding to the pilotin. The 62 residue C-terminus of secretin possesses three putative α-helices (Figure 4A) [22]. If the region consisting of the first two C-terminal helices were deleted, the truncated OutDΔC1 behaves like wild type secretin. Firstly, OutDΔC1 was barely detectable in the absence but was well produced in the presence of pilotin OutS (Figure 4B). Secondly, with pilotin, the mutant secretin was mainly recovered in the outer membrane fractions (Figure 4C). Consistent with the outer membrane location, expression of OutDΔC1 in the presence of OutS results in a rather low level of *pspA* induction. Phage shock protein (psp) response helps to maintain proton motive force in cells under pmf-dissipating stress and is indicative of mislocalization of the secretins in the inner membrane [8], [11]. These results are consistent with the NMR experiments demonstrating that it is the 18 residue C-terminus of OutD which binds tightly to the pilotin. Despite its outer membrane location, OutDΔC1 was unable to restore pectinase secretion in *D. dadantii* Δ*outD* A3559 strain (data not shown) indicating an important functional relevance of the deleted region. Deletion of the extreme C-terminus of secretin resulted in partial stabilization of the truncated OutDΔC2 secretin as judged from the quantity produced in the absence of the pilotin, but prevented its correct targeting to the outer membrane (Figure 4B,C). In the presence of pilotin, the amount of OutDΔC2 was increased indicating that the pilotin can still stabilize and hence interact with the truncated secretin but is not able to target it to the outer membrane. In agreement with this, expression of OutDΔC2 strongly induced *pspA* even in the presence of pilotin. Deletion of the full 62 residue C-terminus of OutD resulted in neither stabilization nor the correct targeting of the truncated secretin OutDΔC3 as judged by low protein content but high *pspA* level (Figure 4B).

### Conclusion

The type II secretin/pilotin complex from *Klebsiella oxytoca* has been imaged by cryo electron microscopy at modest resolution [24]. The secretin subunits form a dodecameric ring with relatively weak radial arms that the authors tentatively assign as the pilotin or pilotin bound to the secretin C-terminus [4], [24]. Comparing the envelope of the complex with that determined by Reichow et al. (2010) of the secretin only [3], the radial arms are located either in the periplasm or in the inner leaflet of the outer membrane. This is the position anticipated from the observation in this work that the pilotin interacts tightly with 18 residues close to the C-terminus of the secretin subunit, so it is entirely plausible that the radial arms seen in the cryo-EM map correspond to the unusual four helix bundle of the type II secretion pilotin bound to the induced C-terminal helix of the secretin subunit.

Recent structural studies have revealed striking structural similarities between components from distant secretion systems and other bacterial cell machines. Besides the expected structural homologies between several conserved components of ancestrally related T2SS and T4P [25]–[27] several other remarkable structural similarities should be mentioned. Notably, the extreme N-terminal N0 domain is shared by secretins from the T2SS, T3SS and T4P but is also structurally related to a domain of lipoprotein DotD from the T4SS, a domain of VgrG from the T6SS and from a TonB dependent receptor FpvA [20], [28]–[30]. Similarly the N1/N2 domains of secretins show a significant structural homology with several ring-forming proteins from the T3SS [20], [31], [32]. It is therefore becoming common to attribute similar function to the proteins or domains of a related structure. Given this background it is remarkable then that the T2SS pilotin described here is profoundly different in architecture to the T3SS pilotin but has similar function. Both the T2SS and the T3SS pilotin bind the extreme C-terminal region of their cognate secretins and this previously unstructured part of the secretin becomes an ordered α-helix on binding. It is therefore remarkable that the corresponding pilotins are different in architecture, one an open β-barrel (T3SS; MxiM) the other an unusual helical bundle (T2SS; OutS). These striking structural differences show that in these systems the pilotins have been evolved independently to play similar roles.

## Materials and Methods

### Plasmid Construction, Expression, Purification and Protein Analysis

The pET-20b(+) plasmid expressing non-lipidated OutS (residues 26 to 132) fused to N-terminal PelB signal peptide has been constructed previously [11] at a manner as the sequence of mature non-lipidated OutS after cleavage by the signal peptidase LepB is: *MD*P^26^VKNT etc. To fuse a C-terminal 6His tag to non-lipidated OutS, *Sal*I site was introduced at the end of *outS* sequence by using the primer (5′-ctt gac gcc atg cgc acc gt**c** **ga**c tga ggg gga agc aac tgc) and the reverse complementary one (mutated bases are underlined). Then, by *Sal*I/*Xho*I digestion the sequence coding for non-lipidated OutS was fused with that coding for 6His. Mutants of OutS were made using Strategene QuikChange and confirmed by sequencing.

To generate OutD truncated derivatives, an *Eco*47III site and V678A substitution were introduced using the primer (5′-gcgcggcgaaggcaacggagcgctggataacaacaccctgc) and the reverse complementary one. This site and naturally existing *Nru*I and *Psi*I sites were used to generate OutDΔC1 (Δ650–678), OutDΔC2 (Δ679–705) and OutDΔC3 (Δ650–705) derivatives. To fuse the C-terminal segments of OutD to GST, the corresponding gene fragments were subcloned from pTdB-OD plasmids expressing either OutD, or OutDΔC1, or OutDΔC2, or OutDΔC3 into pGEX-6P-3 or pGEX-3X vectors in frame with the GST coding sequence.

### Protein Expression, Purification and Analysis

*E. coli* BL21(DE3) strain (Stratagene) was used to produce non-lipidated pilotin (OutS) and GST-secretin (OutD) derivatives. Non-lipidated OutS was released from the periplasm by osmotic shock as described previously [33] and purified by size-exclusion chromatography Superdex S75 10/300 GL (GE Healthcare). The OutD peptide was purified and then released from GST-OutD fusion as described previously [34]. For NMR spectroscopy, uniformly ^15^N- and ^13^C-labeled pilotin and secretin peptides were produced by growing cell cultures in M9 minimal medium that contained ^15^N-ammonium chloride *and* ^13^C-*D-glucose* (Cambridge Isotope Laboratories Inc.) as the sources of nitrogen and carbon, respectively. The 18 residue synthetic secretin peptide (residues 691 to 708 inclusive) was purchased from Generon. Cell membrane fractionation by sucrose density gradient centrifugation was performed as described previously [11] with steady-state cultures of *E. coli* NM522 (Stratagene) expressing OutD derivatives from pTdB-OD and OutS from pACT-S plasmid. The location of outer membrane porins was determined by staining with Coomassie G-250. The position of inner membrane fractions was estimated by immunoblotting with TolA antibodies and NADH oxidase activity. *E. coli* MC3 strain carrying a *pspA-lacZ* fusion [35] was used to estimate miss location of OutD derivatives. To assess functionality of OutD derivatives, complementation assays with *D. dadantii* Δ*outD* A3559 strain were used as previously described [36]. SDS-PAGE and immunoblotting were performed as previously [19]. Anti-OutD rabbit serum was raised against entire OutD purified from a recombinant *E. coli* strain. Anti-TolA serum was kindly provided by J.C. Lazzaroni.

### Crystallisation and Structure Determination

Hampton Research sparse matrix screen was used to search for crystallization conditions. Crystals were grown using hanging drop vapour equilibration using 10 mg/ml OutS and a reservoir of 2 M ammonium sulphate, 2% PEG 400 and 0.1 M HEPES pH 7.5. Vitrification of crystals in liquid nitrogen was achieved using the reservoir solution with 2.1 M ammonium sulphate and augmented with 15–25% glycerol. Data were collected at ESRF ID23-1 and processed using MOSFLM [37] and scaled using SCALA [38]. SAD data were collected from a crystal soaked in 25 mM potassium tetrachloroplatinate (K_2_PtCl_4_) for 4 days. The structure was solved using PHENIX [39] and COOT [40] and refined using the native data at 1.65 Å resolution and non-crystallographic symmetry restraints. The final model comprises 188 amino acid residues and 208 water molecules. DALILITE [41] was used to search for similar structures, CLUSTALW [42] for sequence alignment and JPRED for secondary structure predictions [22]. Crystals of the complex were grown using a 1.0∶1.1 molar ratio of pilotin: secretin peptide and crystallized using a reservoir of 2 M ammonium sulphate, 0.1 M Tris, pH 8.5. Around 100 complex crystals were screened before a well-diffracting reasonable ordered crystal was found. The pilotin/secretin complex was solved by molecular replacement using data collected at DLS I02 and CCP4/PHENIX/COOT. These crystals appear to be P6_5_22 but are most likely twinned P6_5_ with four pilotin molecules in the asymmetric unit. The packing of the molecules is such that there are large solvent channels running through the crystal lattice. These solvent channels appear to have disordered protein present the modelling of which hampers refinement. The disordered regions do not gain clarity if the lower symmetry space group P3_2_ is used (for more details see Table S2).

### Preparation of Proteins for Nmr Spectroscopy and Acquisition of Nmr Spectra

Samples of 0.05 to 0.5 mM labelled proteins in 90% H_2_O, 10% ^2^H_2_O containing 20 mM Tris (pH 7.0) and 150 mM NaCl. All NMR spectra were acquired at 15°C using Bruker Avance 700- and 600-MHz spectrometers. Assignment of ^1^H, ^15^N, and ^13^C resonances of the backbone was achieved by analysis of HNCACB, CBCA (CO)NH triple resonance experiments [43].

### Circular Dichroism

Far-UV CD measurements were made using a Jasco J-715 spectropolarimeter equipped with a PTC-348WI temperature controller. Spectra were recorded in 20 mM Tris, 150 mM NaCl (pH 7.0) at 15°C using 1 mm path length fused silica cuvettes. The spectra are presented as differential absorbance after baseline subtraction. Calculations employed CONTIN, SELCON, and CDSSTR [44].

### Fluorescence Spectroscopy

Fluorescence data were collected using a Jasco FP-6300 Spectrofluorometer. To avoid exciting tyrosyl side chains, an excitation wavelength of 290 nm was used. Emission spectra were recorded at 15°C in steps of 2 nm from 310 to 400 nm. The fluorescence signal at 340 nm was plotted to calculate *K*_d_. Pilotin spectra was measured at 1 µM. 50 µM of secretin peptide prepared in 1 µM pilotin was titrated into pilotin solution (for more information see also Table S1 and reference [45]).

#### Accession codes

Coordinates and structure factor amplitudes have been deposited in the protein databank with the accession codes 3UTK and 3UYM. The sequences of OutS and OutD are available in the UniProt database with accession codes Q01567 and Q01565, respectively.

## Supporting Information

Figure S1 Sequence alignment of pilotins and the C-terminal region of their cognate secretins. The alignment of the pilotins is shown in panel (A) and alignment of the C-terminal region of their cognate secretins in panel (B). The position of the α-helices is indicated by H in the secondary structure row (predicted by Jpred for GspD). Shown are the OutS and OutD homologs of *Dickeya dadantii* (*Erwinia chrysanthemi* 3937), Q01567 and Q01565; *Pectobacterium carotovorum*, C6DAR0 and C6DAQ5; *Escherichia coli* O157:H7, Q7BSV3 and Q9ZGU0; *Klebsiella oxytoca*, P20440 and P15644; *Yersinia mollaretii*, C4S9G3 and C4S9F5; *Serratia odorifera*, D4E1I4 and A8GJQ5. Identical residues are in red, residues similar in character are green. Conserved residues are mapped on to the OutS pilotin structure in Figure 1 of the main text.
(DOC)

Figure S2 Spectroscopic analyses of secretin binding to the pilotin. 2D ^1^H-^15^N HSQC of ^15^N labelled secretin peptide (OutD residues 649–685 and residues 649–710 for the major proteolytic fragment and minor full-length peptide, respectively) in the absence (black) and presence of pilotin (red). The concentration of secretin and pilotin were 50 µM and 100 µM, respectively. Both spectra were acquired using a Bruker 700 MHz at 15°C in buffer comprising 20 mM Tris pH 7.0, 150 mM NaCl and 10% ^2^H_2_O.
(DOC)

Figure S3 Assignment of the backbone amide protons for the C-terminal secretin peptide. The data were acquired at 15°C using peptide in 20 mM Tris at pH 7.0, 150 mM NaCl and 10% ^2^H_2_O and a Bruker 700 MHz.
(DOC)

Figure S4 The C-terminus of the 62 residue secretin peptide (OutD^648–710^) is unstructured. ^1^H-^15^N HSQC spectra of recombinantly produced ^15^N-labelled secretin peptide (70 µM peptide in 20 mM Tris pH 7.0 with 150 mM NaCl at 15°C) acquired using a Bruker 700 MHz spectrophotometer. The low dispersion of the main chain amides reveals the peptide is intrinsically unstructured. The spectra are cleaner than those shown previously (Figure S2 and S3) because the spectra were acquired quickly. During more lengthy experiments the 62 residue secretin peptide is slowly cleaved degrading the quality of the spectra.
(DOC)

Figure S5 3JHNHA spectra of ^15^N-labelled secretin peptide (OutD^680–710^) in the absence (red) and presence (black) of pilotin (OutS). The spectra were acquired using a Bruker 700 MHz spectrophotometer at measured at 15°C. The new peaks, arrowed, have HN-HA coupling constants less than 5 Hz revealing that these residues are helical when bound. The 3JHNHA coupling constant was calculated according to measurement of the diagonal-peak to cross-peak intensity ratio in a 3D ^15^N separated quantitative J-correlation spectra. These spectra show that at least four residues of the secretin peptide become helical on binding to the pilot.
(DOC)

Figure S6 Reducing environment prevents interaction of OutS with the C-terminal peptide of OutD. (A) GST pull-down assay shows that non-lipidated OutS does not bind to the GST-OutD_649–710_ in reducing conditions. Soluble cell extracts of *E. coli* BL21(DE3) producing either GST alone (lane 1) or GST-OutD_649–710_ (lanes 2 and 3) were combined with a periplasmic extract containing non-lipidated OutS, then loaded on Glutathione Agarose for 1 h and washed. The incubations were performed in either TBS (lanes 1 and 2) or TBS with 5 mM DTT (lane 3). Bound proteins were eluted with Laemmli sample buffer, separated by Tricine-SDS-PAGE and either stained (upper panel) or probed with OutS antibodies (lower panel). Asterisk indicates a degradation product of GST-OutD_649–710_. (B) Bacterial two-hybrid assay (Karimova *et al.*, 1998) shows that OutS does not interact with GST-OutD_649–710_ in the reducing conditions of the bacterial cytoplasm. The region coding for mature OutS (residues 26 to 137) was fused to the C-terminus of T18 subunit of Cya (pUT18-OutS) and the region coding for GST-OutD_649–710_ was fused to the C-terminus of T25 subunit of Cya (pKT25-GST-Dct). When pUT18-OutS and pKT25-GST-Dct were coexpressed in *E. coli* DHP1 *cya* strain, the corresponding fusion proteins were well produced as shown by immunoblotting with anti-OutS and anti-GST antibodies, respectively. However, once plated on MacConkey-maltose agar, these bacteria generated white colonies (as did the empty vectors) and not red colonies (as produced by the known interacting OutC/OutC couple which was used as a positive control). T18-OutC is indicated by a dot and T25-GST-Dct by a triangle.
(DOC)

Table S1 Dissociation constants of secretin peptide from pilotin and pilotin mutants determined using fluorescence spectroscopy.
(DOC)

Table S2 Crystallographic data and refinement statistics for pilotin/secretin peptide complex.
(DOC)

## References

[1] Bayan N, Guilvout I, Pugsley AP (2006). Secretins take shape.. Mol Micro.

[2] Collins RF, Frye SA, Kitmitto A, Ford RC, Tonjum T (2004). Structure of the Neisseria meningitidis outer membrane PilQ secretin complex at 12 angstrom resolution.. J Biol Chem.

[3] Reichow SL, Korotkov KV, Hol WGJ, Gonen T (2010). Structure of the cholera toxin secretion channel in its closed state.. Nature Struct Mol Biol.

[4] Chami M, Guilvout I, Gregorini M, Remigy HW, Muller SA (2005). Structural insights into the secretin PulD and its trypsin-resistant core.. J Biol Chem.

[5] Hodgkinson JL, Horsley A, Stabat D, Simon M, Johnson S (2009). Three-dimensional reconstruction of the Shigella T3SS transmembrane regions reveals 12-fold symmetry and novel features throughout.. Nat Struct Mol Biol.

[6] Burghout P, Beckers F, de Wit E, van Boxtel R, Cornelis GR (2004). Role of the pilot protein YscW in the biogenesis of the YscC secretin in Yersinia enterocolitica.. J Bacteriol.

[7] Crago AM, Koronakis V (1998). Salmonella InvG forms a ring-like multimer that requires the InvH lipoprotein for outer membrane localization.. Mol Micro.

[8] Hardie KR, Seydel A, Guilvout I, Pugsley AP (1996). The secretin-specific, chaperone-like protein of the general secretory pathway: Separation of proteolytic protection and piloting functions.. Mol Micro.

[9] Koo J, Tammam S, Ku SY, Sampaleanu LM, Burrows LL (2008). PilF Is an Outer Membrane Lipoprotein Required for Multimerization and Localization of the *Pseudomonas aeruginosa* Type IV Pilus Secretin.. J Bacteriol.

[10] Lario PI, Pfuetzner RA, Frey EA, Creagh L, Haynes C (2005). Structure and biochemical analysis of a secretin pilot protein.. EMBO J.

[11] Shevchik VE, Condemine C (1998). Functional characterization of the *Erwinia chrysanthemi* OutS protein, an element of a type II secretion system.. Microbiology.

[12] Trindade MB, Job V, Contreras-Martel C, Pelicic V, Dessen A (2008). Structure of a widely conserved type IV pilus biogenesis factor that affects the stability of secretin multimers.. J Mol Biol.

[13] Derrick J (2008). A Pilot Sheds Light on Secretin Assembly.. Structure.

[14] Korotkov KV, Gonen T, Hol WGJ (2011). Secretins: dynamic channels for protein transport across membranes.. Trends Biochem Sci.

[15] Okon M, Moraes TF, Lario PI, Creagh AL, Haynes CA (2008). Structural Characterization of the Type-III Pilot-Secretin Complex from *Shigella flexneri*.. Structure.

[16] Golovanov AP, Balasingham S, Tzitzilonis C, Goult BT, Lian LY (2006). The solution structure of a domain from the *Neisseria meningitidis* lipoprotein PiIP reveals a new beta-sandwich fold.. J Mol Biol.

[17] Sandkvist M (2001). Biology of type II secretion.. Mol Microbiol.

[18] Johnson TL, Abendroth J, Hol WGJ, Sandkvist M (2006). Type II secretion: from structure to function.. FEMS Micro Letts.

[19] Shevchik VE, RobertBaudouy J, Condemine G (1997). Specific interaction between OutD, an Erwinia chrysanthemi outer membrane protein of the general secretory pathway, and secreted proteins.. EMBO J.

[20] Korotkov KV, Pardon E, Steyaert J, Hol WGJ (2009). Crystal Structure of the N-Terminal Domain of the Secretin GspD from ETEC Determined with the Assistance of a Nanobody.. Structure.

[21] Guilvout I, Chami M, Engel A, Pugsley AP, Bayan N (2006). Bacterial outer membrane secretin PulD assembles and inserts into the inner membrane in the absence of its pilotin.. EMBO J.

[22] Cole C, Barber JD, Barton GJ (2008). The Jpred 3 secondary structure prediction server.. Nucl Acid Res.

[23] Shoemaker BA, Portman JJ, Wolynes PG (2000). Speeding molecular recognition by using the folding funnel: The fly-casting mechanism.. Proc Natl Acad Sci U S A.

[24] Nouwen N, Ranson N, Saibil H, Wolpensinger B, Engel A (1999). Secretin PulD: Association with pilot PulS, structure, and ion-conducting channel formation.. Proc Natl Acad Sci U S A.

[25] Sampaleanu LM, Bonanno JB, Ayers M, Koo J, Tammam S (2009). Periplasmic Domains of Pseudomonas aeruginosa PilN and PilO Form a Stable Heterodimeric Complex.. J Mol Biol.

[26] Abendroth J, Rice AE, McLuskey K, Bagdasarian M, Hol WGJ (2004). The crystal structure of the periplasmic domain of the type II secretion system protein EpsM from Vibrio cholerae: The simplest version of the ferredoxin fold.. J Mol Biol.

[27] Abendroth J, Kreger AC, Hol WGJ (2009). The dimer formed by the periplasmic domain of EpsL from the Type 2 Secretion System of Vibrio parahaemolyticus.. J Str Biol.

[28] Nakano N, Kubori T, Kinoshita M, Imada K, Nagai H (2010). Crystal Structure of Legionella DotD: Insights into the Relationship between Type IVB and Type II/III Secretion Systems.. PLOS Pathog.

[29] Leiman PG, Basler M, Ramagopal UA, Bonanno JB, Sauder JM (2009). Type VI secretion apparatus and phage tail-associated protein complexes share a common evolutionary origin.. Proc Natl Acad Sci U S A.

[30] Garcia-Herrero A, Vogel HJ (2005). Nuclear magnetic resonance solution structure of the periplasmic signalling domain of the TonB-dependent outer membrane transporter FecA from Escherichia coli.. Mol Micro.

[31] Yip CK, Kimbrough TG, Felise HB, Vuckovic M, Thomas NA (2005). Structural characterization of the molecular platform for type III secretion system assembly.. Nature.

[32] Worrall LJ, Vuckovic M, Strynadka NCJ (2010). Crystal structure of the C-terminal domain of the Salmonella type III secretion system export apparatus protein InvA.. Protein Sci.

[33] Fries M, Ihrig J, Brocklehurst K, Shevchik VE, Pickersgill RW (2007). Molecular basis of the activity of the phytopathogen pectin methylesterase.. EMBO J.

[34] Login FH, Shevchik VE (2006). The single transmembrane segment drives self-assembly of OutC and the formation of a functional type II secretion system in *Erwinia chrysanthemi*.. J Biol Chem.

[35] Bergler H, Abraham D, Aschauer H, Turnowsky F (1994). Inhibition of lipid biosynthesis induces the expression of the pspa gene.. Microbiology.

[36] Bouley J, Condemine G, Shevchik VE (2001). The PDZ domain of OutC and the N-terminal region of OutD determine the secretion specificity of the type II out pathway of *Erwinia chrysanthemi*.. J Mol Biol.

[37] Leslie AGW (2006). The integration of macromolecular diffraction data.. Acta Cryst.

[38] Evans P (2006). Scaling and assessment of data quality.. Acta Cryst.

[39] Adams PD, Afonine PV, Bunkoczi G, Chen VB, Davis IW (2010). PHENIX: a comprehensive Python-based system for macromolecular structure solution.. Acta Cryst.

[40] Emsley P, Lohkamp B, Scott WG, Cowtan K (2010). Features and development of Coot.. Acta Cryst.

[41] Holm L, Kaariainen S, Rosenstrom P, Schenkel A (2008). Searching protein structure databases with DaliLite v.3.. Bioinformatics.

[42] Larkin MA, Blackshields G, Brown NP, Chenna R, McGettigan PA (2007). Clustal W and clustal X version 2.0.. Bioinformatics.

[43] Bax A, Grishaev A (2005). Weak alignment NMR: a hawk-eyed view of biomolecular structure.. Curr Opin Struct Biol.

[44] Sreerama N, Venyaminov SY, Woody RW (2000). Estimation of protein secondary structure from circular dichroism spectra: Inclusion of denatured proteins with native proteins in the analysis.. Anal Biochem.

[45] Martin SR, Schilstra MJ (2008). Circular dichroism and its application to the study of biomolecules.. Biophysical Tools for Biologists: Vol 1 in Vitro Techniques.

[46] Weiss MS (2001). Global indicators of X-ray data quality.. J Appl Cryst.

