# Supplementary material for: Structural and Functional Insights into the Pilotin-Secretin Complex of the Type II Secretion System
Source: PLoS Pathog. 2012 Feb 9;8(2):e1002531. doi: 10.1371/journal.ppat.1002531 (PMC3276575; doi:10.1371/journal.ppat.1002531)
Supplement: Figure S1 — Sequence alignment of pilotins and the C-terminal region of their cognate secretins. The alignment of the pilotins is shown in panel (A) and alignment of the C-terminal region of their cognate secretins in panel (B). The position of the α-helices is indicated by H in the secondary structure row (predicted by Jpred for GspD). Shown are the OutS and OutD homologs of Dickeya dadantii (Erwinia chrysanthemi 3937), Q01567 and Q01565; Pectobacterium carotovorum, C6DAR0 and C6DAQ5; Escherichia coli O157:H7, Q7BSV3 and Q9ZGU0; Klebsiella oxytoca, P20440 and P15644; Yersinia mollaretii, C4S9G3 and C4S9F5; Serratia odorifera, D4E1I4 and A8GJQ5. Identical residues are in red, residues similar in character are green. Conserved residues are mapped on to the OutS pilotin structure in Figure 1 of the main text. (DOC) [file ppat.1002531.s001.doc]

**A**

**Sec. str. -------HHHHHHHHHHHHHHH----- -------- -------HHHHHHHHHHHHHHHHHHHHH------**

**10 20 30 40 50 60**

**| | | | | |**

**OutS_Ddad ---MHVSSLKVVLFGVCCLSLAACQTP--APVKNTAS--RSAASVPANEQISQLASLVAASKYLRVQCERSDL**

**OutS_Pcar ---MSLSLSKFSALTLLCVTLAGCQQTGTSAHKGTVAGQSSAVTVSPNDQLNQLSSLVAATRYLKSKCNRSDL**

**GspS_Ecol MMGNILKKLNCIASLLVLVTISGCHQS--PSIHKQAT-------VPPSEQLEQMASIVSATRYLKMRCNRSDL**

**PulS_Koxy ------MRNFILFPMMAVVLLSGCQQN--RPTTLSPA-------VSGQAQLEQLASVAAGARYLKNKCNRSDL**

**GspS_Ymol ----MLSTTRKLFWFLPLVLLTGCQQP-MNKTIKPTA-------Q---QQIKQLSALVAGAHYLQKNCQRAEV**

**GspS_Spro ---MQRMLRSFSFAAAAVVLLAGCQQTALQPQTQPSL-------T---AQLDQLSALLAGSQFLRQHCARTDI**

**Sec. str. --HHHHHHHHHHHHHH----H HH----HHHHHHHHHHHHH----HHHHHHHHHHH-HHHHHHHH---**

**70 80 90 100 110 120 130**

**| | | | | | |**

**OutS_Ddad PDDGTILKTAVNVAVQKGWDT--GRYQSLPQLSENLYQGLLKDGTPKATQCSSFNRTMTPFLDAMRTVR--**

**OutS_Pcar PDDATVMNVALTVAKQRGWNV--ASYQALPQRSESLYQGLLKDSTPKETQCSEFNRTLTPFIDAIRSRG--**

**GspS_Ecol PDEQSILNVANRIAIGKGWQS--LTQEDIRKHSDDIYVRLTRDSTPEYIKCREFNRRLVPFIGELLARGRG**

**PulS_Koxy PADEAINRAAINVGKKRGWAN--IDANLLSQRSAQLYQQLQQDSTPEATKCSQFNRQLAPFIDSLRDNK--**

**GspS_Ymol PDEAVLLKTARSLAASRHWDTRAPAYKLLGEQSQARYQALVKENETDKSMCTELNLLMVDFVDEAQRNIK**

**GspS_Spro PDDASLQRSAIGMAQQRGWNTQPAEYRQLPVRAQQRYQQLQQDGTPLQQKCAALNTSTARFIAAAQSDARQ**

**B**

**Sec. str. ---------HHHHHHHH---HHHHHHH------ ---------- -----HHHHHHHHHHHH------**

**650 660 670 680 690 700 710**

**| | | | | | |**

**OutD_Ddad TIIRDPGQFQEASINKYRSFNNEQQQQRGEGNG---VLDNNTLRLS-GGNTYTFRQVQSSISDFYKPEGR**

**OutD_Pcar SIIRDRSQYQSASASKYHSFNAEEEKQREANGGKANLLDNDLLRLPEGGNAYTFRQVQSSIVAFYPAGGK**

**GspD_Ecol TIIRERDGFRHASAEKYQSFNQEQVQSRGKETT-ALTLNEEQLRLSPDQDDTAFRKVKAAIAAFYAQEM-**

**PulD_Koxy TVIRDRDEYRQASSGQYTAFNDAQSKQRGKENND-AMLNQDLLEIYPRQDTAAFRQVSAAIDAFNLGGNL**

**GspD_Ymol TIIREQDSYTDLSEQRLDKFQQEQNRDRPASGQ---RINENLNNIL--SDGRSLQDLRNDVSAFYSKGA-**

**GspD_Spro TVIRSTEEYAQESMRKAARFRSLDAQPLPLKTH----AEAQLDRVG-STENGTFQRIQRQIDAFYPRGEL**

**Figure S1.** Sequence alignment of pilotins and the C-terminal region of their cognate secretins. The alignment of the pilotins is shown in panel (A) and alignment of the C-terminal region of their cognate secretins in panel (B). The position of the α-helices is indicated by H in the secondary structure row (predicted by Jpred for GspD). Shown are the OutS and OutD homologs of *Dickeya* *dadantii* (*Erwinia* *chrysanthemi* 3937), Q01567 and Q01565; *Pectobacterium* *carotovorum*, C6DAR0 and C6DAQ5; *Escherichia* *coli* O157:H7, Q7BSV3 and Q9ZGU0; *Klebsiella* *oxytoca*, P20440 and P15644; *Yersinia* *mollaretii*, C4S9G3 and C4S9F5; *Serratia* *odorifera*, D4E1I4 and A8GJQ5. Identical residues are in red, residues similar in character are green. Conserved residues are mapped on to the OutS pilotin structure in Figure 1 of the main text.
