# Supplementary material for: Structural and Functional Insights into the Pilotin-Secretin Complex of the Type II Secretion System
Source: PLoS Pathog. 2012 Feb 9;8(2):e1002531. doi: 10.1371/journal.ppat.1002531 (PMC3276575; doi:10.1371/journal.ppat.1002531)
Supplement: Figure S2 — Spectroscopic analyses of secretin binding to the pilotin. 2D 1H-15N HSQC of 15N labelled secretin peptide (OutD residues 649–685 and residues 649–710 for the major proteolytic fragment and minor full-length peptide, respectively) in the absence (black) and presence of pilotin (red). The concentration of secretin and pilotin were 50 µM and 100 µM, respectively. Both spectra were acquired using a Bruker 700 MHz at 15°C in buffer comprising 20 mM Tris pH 7.0, 150 mM NaCl and 10% 2H2O. (DOC) [file ppat.1002531.s002.doc]

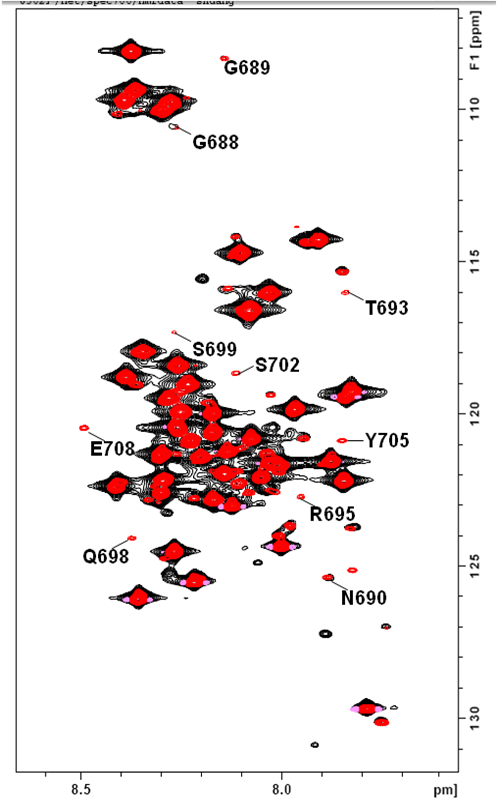


^15^N (ppm)

^1^H (ppm)

**Figure S2.** Spectroscopic analyses of secretin binding to the pilotin. 2D ^1^H-^15^N HSQC of ^15^N labelled secretin peptide (OutD residues 649-685 and residues 649-710 for the major proteolytic fragment and minor full-length peptide, respectively) in the absence (black) and presence of pilotin (red). The concentration of secretin and pilotin were 50 μM and 100 μM, respectively. Both spectra were acquired using a Bruker 700MHz at 15 ºC in buffer comprising 20mM Tris pH 7.0, 150mM NaCl and 10% ^2^H_2_O.

B
