# Supplementary material for: Structural and Functional Insights into the Pilotin-Secretin Complex of the Type II Secretion System
Source: PLoS Pathog. 2012 Feb 9;8(2):e1002531. doi: 10.1371/journal.ppat.1002531 (PMC3276575; doi:10.1371/journal.ppat.1002531)
Supplement: Figure S3 — Assignment of the backbone amide protons for the C-terminal secretin peptide. The data were acquired at 15°C using peptide in 20 mM Tris at pH 7.0, 150 mM NaCl and 10% 2H2O and a Bruker 700 MHz. (DOC) [file ppat.1002531.s003.doc]

^15^N (ppm)

^1^H (ppm)

**Figure S3.** Assignment of the backbone amide protons for the C-terminal secretin peptide (a part of Figure S2A is expanded along x-axis to facilitate labelling). The data were acquired at 15 ⁰C using peptide in 20mM Tris at pH 7.0, 150mM NaCl and 10% ^2^H_2_O and a Bruker 700MHz.
