# Supplementary material for: Structural and Functional Insights into the Pilotin-Secretin Complex of the Type II Secretion System
Source: PLoS Pathog. 2012 Feb 9;8(2):e1002531. doi: 10.1371/journal.ppat.1002531 (PMC3276575; doi:10.1371/journal.ppat.1002531)
Supplement: Figure S4 — The C-terminus of the 62 residue secretin peptide (OutD648–710) is unstructured. 1H-15N HSQC spectra of recombinantly produced 15N-labelled secretin peptide (70 µM peptide in 20 mM Tris pH 7.0 with 150 mM NaCl at 15°C) acquired using a Bruker 700 MHz spectrophotometer. The low dispersion of the main chain amides reveals the peptide is intrinsically unstructured. The spectra are cleaner than those shown previously (Figure S2 and S3) because the spectra were acquired quickly. During more lengthy experiments the 62 residue secretin peptide is slowly cleaved degrading the quality of the spectra. (DOC) [file ppat.1002531.s004.doc]

**
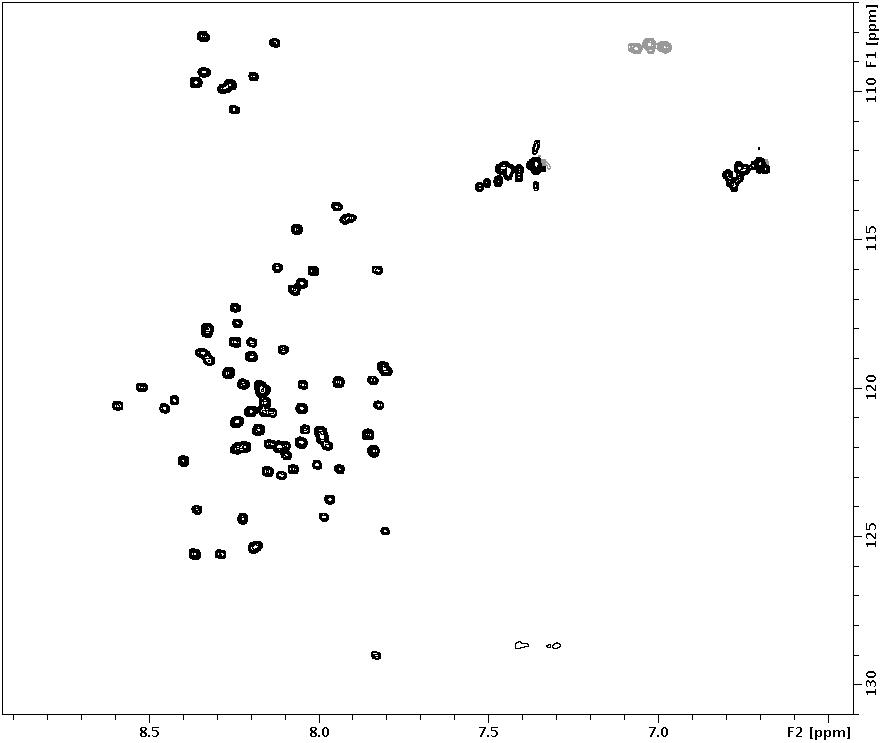
**

^15^N (ppm)

^1^H (ppm)

**Figure S4.** The C-terminus of the 62 residue secretin peptide (OutD^648-710^) is unstructured. ^1^H-^15^N HSQC spectra of recombinantly produced ^15^N-labelled secretin peptide (70 μM peptide in 20mM Tris pH7.0 with 150mM NaCl at 15 ºC) acquired using a Bruker 700MHz spectrophotometer. The low dispersion of the main chain amides reveals the peptide is intrinsically unstructured. The spectra are cleaner than those shown previously (Figure S2 and S3) because the spectra were acquired quickly. During more lengthy experiments the 62 residue secretin peptide is slowly cleaved degrading the quality of the spectra.
