# Supplementary material for: Structural and Functional Insights into the Pilotin-Secretin Complex of the Type II Secretion System
Source: PLoS Pathog. 2012 Feb 9;8(2):e1002531. doi: 10.1371/journal.ppat.1002531 (PMC3276575; doi:10.1371/journal.ppat.1002531)
Supplement: Figure S5 — 3JHNHA spectra of 15N-labelled secretin peptide (OutD680–710) in the absence (red) and presence (black) of pilotin (OutS). The spectra were acquired using a Bruker 700 MHz spectrophotometer at measured at 15°C. The new peaks, arrowed, have HN-HA coupling constants less than 5 Hz revealing that these residues are helical when bound. The 3JHNHA coupling constant was calculated according to measurement of the diagonal-peak to cross-peak intensity ratio in a 3D 15N separated quantitative J-correlation spectra. These spectra show that at least four residues of the secretin peptide become helical on binding to the pilot. (DOC) [file ppat.1002531.s005.doc]

^15^N (ppm)

^1^H (ppm)

**Figure S5.** 3JHNHA spectra of ^15^N-labelled secretin peptide (OutD^680-710^) in the absence (red) and presence (black) of pilotin (OutS). The spectra were acquired using a Bruker 700MHz spectrophotometer at measured at 15ºC. The new peaks, arrowed, have HN-HA coupling constants less than 5 Hz revealing that these residues are helical when bound. The 3JHNHA coupling constant was calculated according to measurement of the diagonal-peak to cross-peak intensity ratio in a 3D ^15^N separated quantitative J-correlation spectra. These spectra show that at least four residues of the secretin peptide become helical on binding to the pilot.
