# Supplementary material for: Structural and Functional Insights into the Pilotin-Secretin Complex of the Type II Secretion System
Source: PLoS Pathog. 2012 Feb 9;8(2):e1002531. doi: 10.1371/journal.ppat.1002531 (PMC3276575; doi:10.1371/journal.ppat.1002531)
Supplement: Figure S6 — Reducing environment prevents interaction of OutS with the C-terminal peptide of OutD. (A) GST pull-down assay shows that non-lipidated OutS does not bind to the GST-OutD649–710 in reducing conditions. Soluble cell extracts of E. coli BL21(DE3) producing either GST alone (lane 1) or GST-OutD649–710 (lanes 2 and 3) were combined with a periplasmic extract containing non-lipidated OutS, then loaded on Glutathione Agarose for 1 h and washed. The incubations were performed in either TBS (lanes 1 and 2) or TBS with 5 mM DTT (lane 3). Bound proteins were eluted with Laemmli sample buffer, separated by Tricine-SDS-PAGE and either stained (upper panel) or probed with OutS antibodies (lower panel). Asterisk indicates a degradation product of GST-OutD649–710. (B) Bacterial two-hybrid assay (Karimova et al., 1998) shows that OutS does not interact with GST-OutD649–710 in the reducing conditions of the bacterial cytoplasm. The region coding for mature OutS (residues 26 to 137) was fused to the C-terminus of T18 subunit of Cya (pUT18-OutS) and the region coding for GST-OutD649–710 was fused to the C-terminus of T25 subunit of Cya (pKT25-GST-Dct). When pUT18-OutS and pKT25-GST-Dct were coexpressed in E. coli DHP1 cya strain, the corresponding fusion proteins were well produced as shown by immunoblotting with anti-OutS and anti-GST antibodies, respectively. However, once plated on MacConkey-maltose agar, these bacteria generated white colonies (as did the empty vectors) and not red colonies (as produced by the known interacting OutC/OutC couple which was used as a positive control). T18-OutC is indicated by a dot and T25-GST-Dct by a triangle. (DOC) [file ppat.1002531.s006.doc]

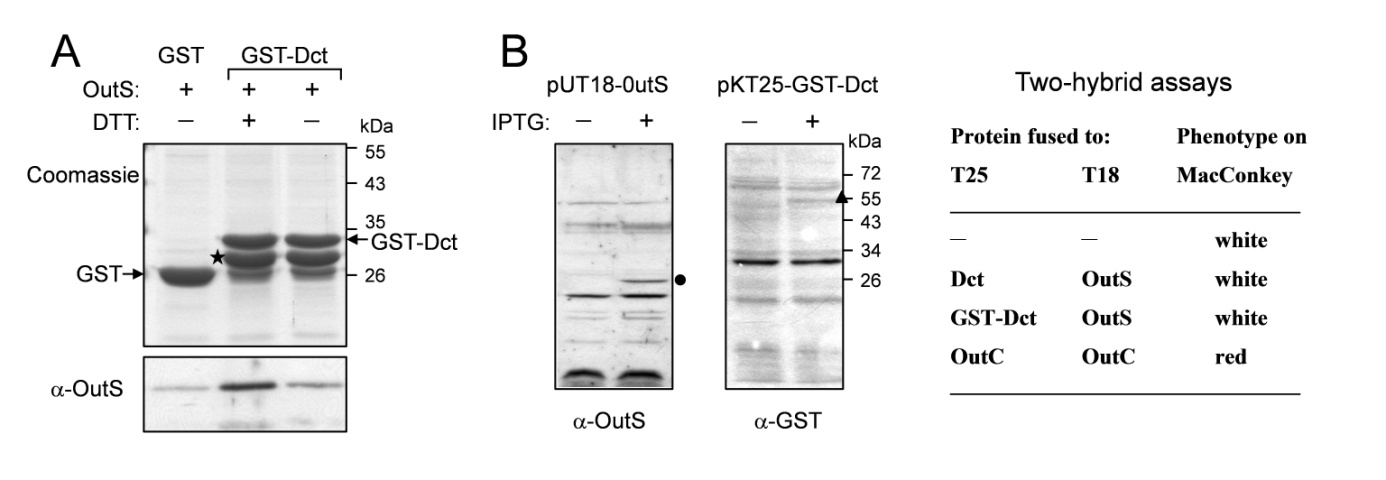


**Figure S6**. Reducing environment prevents interaction of OutS with the C-terminal peptide of OutD. (A) GST pull-down assay shows that non-lipidated OutS does not bind to the GST-OutD_649-710_ in reducing conditions. Soluble cell extracts of *E. coli* BL21(DE3) producing either GST alone (lane 1) or GST-OutD_649-710_ (lanes 2 and 3) were combined with a periplasmic extract containing non-lipidated OutS, then loaded on Glutathione Agarose for 1 h and washed. The incubations were performed in either TBS (lanes 1 and 2) or TBS with 5 mM DTT (lane 3). Bound proteins were eluted with Laemmli sample buffer, separated by Tricine-SDS-PAGE and either stained (upper panel) or probed with OutS antibodies (lower panel). Asterisk indicates a degradation product of GST-OutD_649-710_. (B) Bacterial two-hybrid assay (Karimova *et al*., 1998) shows that OutS does not interact with GST-OutD_649-710_ in the reducing conditions of the bacterial cytoplasm. The region coding for mature OutS (residues 26 to 137) was fused to the C-terminus of T18 subunit of Cya (pUT18-OutS) and the region coding for GST-OutD_649-710_ was fused to the C-terminus of T25 subunit of Cya (pKT25-GST-Dct). When pUT18-OutS and pKT25-GST-Dct were coexpressed in *E. coli* DHP1 *cya* strain, the coresponding fusion proteins were well produced as shown by immunoblotting with anti-OutS and anti-GST antibodies, respectively. However, once plated on MacConkey-maltose agar, these bacteria generated white colonies (as did the empty vectors) and not red colonies (as produced by the known interacting OutC/OutC couple which was used as a positive control). T18-OutC is indicated by a dot and T25-GST-Dct by a triangle.
