# Supplementary material for: Structural and Functional Insights into the Pilotin-Secretin Complex of the Type II Secretion System
Source: PLoS Pathog. 2012 Feb 9;8(2):e1002531. doi: 10.1371/journal.ppat.1002531 (PMC3276575; doi:10.1371/journal.ppat.1002531)
Supplement: Table S1 — Dissociation constants of secretin peptide from pilotin and pilotin mutants determined using fluorescence spectroscopy. (DOC) [file ppat.1002531.s007.doc]

**Table S1.** Dissociation constants of secretin peptide from pilotin and pilotin mutants determined using fluorescence spectroscopy.

| Protein | *K_d_* |
| --- | --- |
| OutS | 0.055± 0.020 μM |
| OutS_Q114A | 1.1±0.35 μM |
| OutS_L100A | 2.1±0.5 μM |
| OutS_L96A | 5.3±1.2 μM |
| OutS_S49R | >200 μM |

The *K_d_* was determined by fitting the following equation:

[PS] = **{** (*K_d_*+[P_0_]+[S_0_]) – {(*K_d_*+[P_0_]+[S_0_])^2^ – 4[P_0_][S_0_]}^1/2^ **}**/ 2

where [PS] is the concentration of the pilotin/secretin complex, [P_0_] is the total concentration of the pilotin (1 μM), [S_0_] is the total concentration of the secretin peptide, and *K_d_* is the dissociation constant. The observed fluorescence signal is then given by: F = F_PS_[PS] + F_P_[P]; as there are no tryptophans in the secretin peptide. [P] = [P_0_] – [PS].
