# Supplementary material for: Structural and Functional Insights into the Pilotin-Secretin Complex of the Type II Secretion System
Source: PLoS Pathog. 2012 Feb 9;8(2):e1002531. doi: 10.1371/journal.ppat.1002531 (PMC3276575; doi:10.1371/journal.ppat.1002531)
Supplement: Table S2 — Crystallographic data and refinement statistics for pilotin/secretin peptide complex. (DOC) [file ppat.1002531.s008.doc]

| **Table S2.** Crystallographic data and refinement statistics for pilotin/secretin peptide complex | |
| --- | --- |
| *Data Collection* |  |
| Space group | P6_5_^†^ |
| Cell edges (Å) | *a =91.9, c=141.6* |
| Molecules per asymmetric unit | 4 |
| Wavelength (Å) | 1.0718 |
| Resolution (Å) | 46.55–2.00 (2.16–2.00) ^a^ |
| Number of unique reflections | 46764 (6813) ^a^ |
| Multiplicity | 5.4 (5.4) ^a^ |
| Completeness (%) | 99.8 (99.8) ^a^ |
| R_merge_ (%)^b^ | 0.098 (0.354)^a^ |
| Mean I/sigma (I) | 13.3 (3.4) ^a^ |
| R_pim_ (%)^c^ | 0.046 (0.169) ^a^ |
| Wilson B-factor ( Å^2^) | 17.1 |
| *Refinement* |  |
| Resolution limits (Å) | 23.22 – 2.00 |
| Reflections (work/test) | 44205/2377 |
| R-factor/R-free^d^ (%) | 0.243/0.279 |
| rmsd bond(Å) /angle (°) | 0.006/0.949 |
| Number of protein (solvent) atoms | 3401 (996)^‡^ |
| Ramachandran plot statistics (%) |  |
| Residues in most favoured regions | 93.1% |
| Residues in additional allowed regions | 6.9% |
| ^a^The parameter values for the range 2.16-2.00Å are given in parentheses. ^b^ R_merge_ = Σ_hkl_ Σ_i_ |I_i_ - <I> | /_Σ_hkl_ ΣI_i_, where I_i_ is the intensity of the i^th^ observation, <I> is the mean intensity of the reflection, and the summations extend over all unique reflections (hkl) and all equivalents (i), respectively. ^c^ R_pim_ = Σ_hkl_ [n/(n-1)]^1/2^ Σ_i_ |I_i_(hkl) - <I(hkl) >| / Σ_hkl_ Σ_i_ I_i_(hkl), where n is the multiplicity, other variables as defined for R_merge_ [46]. ^d^R-factor = Σ_hkl_ |F_o_ – F_c_ | / Σ_hkl_ F_o_, where F_o_ and F_c_ represent the observed and calculated structure factors, respectively. The R-Factor is calculated using 95% of the data included in refinement and R-free the 5% excluded. ^†^The space group appears to be P6_5_22, but twinning is indicated in the cumulative intensity distribution. The data can be more successfully refined in P6_5_ with the twinning operator: h, -h-k, -l. Using the lower symmetry space group, P3_2_ with twinning operators, does not further facilitate refinement of the structure. ^‡^The disordered protein detected in the solvent channels was modelled using water molecules which is why the number of solvent atoms is high. The presence of disordered density in the solvent channel is far from ideal and the density became no clearer in lower symmetry space groups, but the original map and the simulated annealed omit map shown in Figure 3C does provide evidence for the structure of the bound secretin peptide. | |
